# Characterization and Comparison of Extended-Spectrum β-Lactamase (ESBL) Resistance Genotypes and Population Structure of *Escherichia coli* Isolated from Franklin's Gulls (*Leucophaeus pipixcan*) and Humans in Chile

**DOI:** 10.1371/journal.pone.0076150

**Published:** 2013-09-30

**Authors:** Jorge Hernandez, Anders Johansson, Johan Stedt, Stina Bengtsson, Aleksandra Porczak, Susanne Granholm, Daniel González-Acuña, Björn Olsen, Jonas Bonnedahl, Mirva Drobni

**Affiliations:** 1 Section of Clinical Microbiology and Infectious Diseases, Department of Medical Sciences, Uppsala University, Uppsala, Sweden; 2 Department of Clinical Microbiology, Umeå University, Umeå, Sweden; 3 Centre for Ecology and Evolution in Microbial Model Systems, School of Natural Sciences, Linnaeus University, Kalmar, Sweden; 4 Clinical Microbiology, Central Hospital, Växjö, Sweden; 5 Departamento de Ciencias Pecuarias, Facultad de Ciencias Veterinarias, Universidad de Concepción, Concepción, Chile; 6 Department of Infectious Diseases, Kalmar County Hospital, Kalmar, Sweden; Institut National de la Recherche Agronomique, France

## Abstract

We investigated the general level of antibiotic resistance with further analysis of extended-spectrum beta-lactamase (ESBL) prevalence, as well as the population structure of *E. coli* in fecal flora of humans and Franklin’s gulls (*Leucophaeus pipixcan*) in central parts of Chile. We found a surprisingly high carriage rate of ESBL-producing *E. coli* among the gulls 112/372 (30.1%) as compared to the human population 6/49 (12.2%.) Several of the *E. coli* sequence types (STs) identified in birds have previously been reported as Multi Drug Resistant (MDR) human pathogens including the ability to produce ESBLs. This means that not only commensal flora is shared between birds and humans but also STs with pathogenic potential. Given the migratory behavior of Franklin’s gulls, they and other migratory species, may be a part of ESBL dissemination in the environment and over great geographic distances. Apart from keeping the antibiotic use low, breaking the transmission chains between the environment and humans must be a priority to hinder the dissemination of resistance.

## Introduction

Extended spectrum β-lactamases of CTX-M type causing antibiotic resistance in gram-negative bacteria emerged at several locations worldwide in the 1980s [1], and have since become a major threat to modern medicine. Spread of CTX-M type ESBL was in many parts of the world preceded by an initial spread of SHV and TEM ESBL type, but in South America these did not have the same early dissemination [2]. Instead CTX-M ESBLs emerged early and are now widely distributed in countries such as Argentina and Brazil from which most reports are available [2], [3], [4]. In Chile, the usage of antibiotics is large and uncontrolled, self-prescription is common, and antibiotics are sold over the counter [2]. Fish farming also consumes vast amounts of antibiotics and Chile is among the top consumers of antibiotics in the world [5]. Reports have confirmed an increase in bacterial antibiotic resistance in demersal and pelagic fish captured at the Chilean coast [6], [7]. However, reports on CTX-M dissemination in Chile are still few [8], and the occurrence of these bacteria among humans and in the environment is unclear.

Clinical and environmental dissemination of CTX-M ESBLs seem to be linked, and several reports have shown similarities between clinical human samples and wild avian samples [9], [10]. Previous studies have often focused on avian species with close human contact, mainly gulls of different species that may be migratory or non-migratory depending on the specific population, to investigate the dissemination potential. The Chilean coast is visited every austral summer by hundreds of thousands of Franklin’s gulls *Leucophaeus pipixcan*. These gulls are mainly restricted to the coast and have an opportunistic feeding behavior, seeking their food along the shoreline, offshore, but also in human garbage. After their wintering period in Chile, they return to the central parts of Canada where they breed. Hence the contact with humans and a potential to spread resistant bacteria both within large bird colonies locally and through migration between continents make comparisons of antibiotic resistance markers in fecal bacteria in humans and gulls interesting.

CTX-M ESBLs have high dissemination potential in bacterial populations due to the location of such resistance genes on transferable plasmids. In epidemiological investigations of plasmid bound resistance, the typing of replicons, which are genetic structures encoding replication control mechanisms, has been well described and frequently used [11]. It is believed that features of resistance plasmid replicons define their dissemination capabilities. The most widespread plasmid replicon variants (or Inc-families) are in the IncF family (FIA, FIB, FIC and FII), a heterogeneous group of plasmids commonly found in collections of *Escherichia coli* [12], [13]. These replicon types may also be found together in the same plasmids, then designated multireplicons, one very common combination being FII-FIB [14]. A PCR-based replicon typing (PBRT) scheme identifying 18 of the 27 major plasmid incompatibility groups (Inc) found in *Enterobacteriaceae* [15] was developed and introduced by Carattoli *et al.* and is now widely used [16].

When investigating the zoonotic features and dissemination potential of *E. coli* between humans and the environment, an understanding of the population genetics of the bacterium is highly important. Using multi locus sequence typing (MLST), it has previously been shown that *E. coli* sequence types (STs) that are common among humans also occur in avians, precluding a clear separation of human and environmental *E. coli* populations [9], [10], [17].

Here we present a study of two species that live in close proximity to each other: humans and Franklin's gulls at the Chilean coast. Using a large material of both humans and Franklin’s gulls, we were able to compare their *E. coli* populations (as determined by MLST) as well as resistance genotypes (through replicon and resistance genotyping) This work illustrates the zoonotic potential of *E. coli* and that there has been an exchange of antibiotic resistance genes between birds and humans.

## Materials and Methods

### Collection of Franklin’s gull samples

In January 2009, we collected 370 fresh fecal samples from adult Franklin’s gulls at their wintering sites in Chile, South America. 197 samples were collected at the Aconcagua river delta and 173 samples near the Bio-Bio river delta. The distance between the Aconcagua and the Bio-Bio rivers is approximately 600 km and both rivers run through highly populated regions before they enter the Pacific Ocean at the city of Concón and Talcahuano, respectively. Avian fecal material was submerged in bacterial storage medium (Luria broth: BD, Sparks, USA, in phosphate-buffered saline with the addition of 0.45% Na-citrate, 0.1% MgSO4, 1% (NH4) 2SO4 and 4.4% glycerol). After sampling, samples were transported and stored at −70°C for later examination.

### Collection of human clinical samples

In March 2010, 49 human fecal samples from randomly selected volunteers (28 females and 21 males) aged 0 to 82 years (mean 36.5; median 38) were collected. The volunteers were primary health care (PHC) patients at 18 different centers in 5 different counties visiting for non-infection related causes. All centers were located in the San Antonio area between the Aconcagua river delta in Con-Con and the Maipo river delta in Llo-Lleo. Samples were taken at home and patients were instructed to cool the samples before deposit them at the PHC facility the same or the following day. Upon delivery at the PHC center the samples were submerged in bacterial storage medium, transported and stored as described above for avian samples.

### Ethics statement

The human sampling took place in San Antonio, Chile in cooperation with Hospital Claudio Vicuña, San Antonio, Chile. The ethics committe at the Hospital Claudio Vicuña, San Antonio Chile has issued a formal written waiver which state as follows (English translation):

"After the acceptance of the Director of the Hospital Claudio Vicuña in 2010 the project "Presence of Beta-lactamase producing bacteria in Birds and Humans in Chile" with the applicant Doctor Sr: Jorge Hernandez, University of Linneus and University of Uppsala, Hospital Director Dr. Milton Egaña, Claudio Vicuña authorizes and assumes the ethical responsibility of the establishing and the proceeding to human fecal sampling of the population of San Antonio consulting primary care centers, since that the procedure was adjusted to the principles and protocols in place at the Hospital Ethics Committee, i.e., in free decision with informed consent and under the commitment to withhold personal data of people who voluntarily agree to participate in the project". The participation was voluntarily and a written informed consent was collected from every participant. If the participant was below the age of 18, a written informed consent was collected from a parent.

A written permission to perform the gull sampling on the sites visited (only public places) was issued from the department of Agriculture, “Ministerio de Agricultura, Gobierno de Chile”. Since the fecal samples were taken from the ground there was no interaction with the gulls at all.

### Random selection of *E. coli* isolates from human and avian samples

Each fecal sample was inoculated on UriSelect 4 agar plates (Bio-Rad Laboratories, Marnes-La-Coquette, France) for isolation of putative *E. coli*. When growth was present, one randomly selected colony was isolated from each sample. *E. coli* species identity was confirmed by conventional biochemical testing.

### Antibiotic susceptibility analysis

The antibiotic susceptibility of the *E. coli* isolates was tested against a set of antibiotic agents including tetracycline, ampicillin, streptomycin, chloramphenicol, nalidixic acid, cefadroxil, tigecycline, Trimethoprim/sulfamethoxazole, nitrofurantoin and mecillinam. These antibiotics were selected to represent commonly used agents against *E. coli* infections in human and veterinary medicine. Resistance was determined by antibiotic disk diffusion (discs from Oxoid, Basingstoke, UK) on Mueller-Hinton agar in accordance with recommendations from EUCAST (The European Committee on Antimicrobial Susceptibility Testing), using *E. coli* ATCC 25922 as reference strain in all assessments. For antibiotics lacking EUCAST-defined breakpoints for *E. coli* (tetracycline and streptomycin), the NRI method [18] also used by EUCAST, was implemented to define a local breakpoint.

### Isolation of ESBL-producing bacteria

All fecal samples of human or avian origin were also enriched in brain heart infusion broth (Becton Dickinson, Franklin Lakes, NJ, USA) supplemented with vancomycin (16 mg/L, ICN Biomedicals Inc. Aurora, OH, USA)) for 18h at 37°C, and subsequently inoculated and cultured overnight at 37°C on chromID™ ESBL plates (bioMérieux, Marcy ĹEtoile, France), according to manufacturer’s instructions. Colonies were isolated and species identity confirmed by biochemical testing. ESBL production was confirmed by cefpodoxime/cefpodoxime+clavulanic acid double disk test (MAST Diagnostics, Bootle, UK), before genetic characterisation.

### Genetic characterisation of ESBL variants

The presence of different *bla*_CTX-M_ genotypes was determined using a multiplex real-time PCR protocol [19] that display group designation (CTX-M-1/-2/-8/25 and -9) of *bla*_CTX-M_ positive isolates. A subset of ESBL-producing isolates, that was also investigated by MLST (see below), was further characterised by nucleotide sequencing of the CTX-M-1 and CTX-M-9 genes as described previously [20], [21], and for the presence of *bla*_TEM_ and *bla*_SHV_ genes using primers from Pitout et al [22] and a previously described SYBR®Green based real-time PCR protocol [10]. The *bla*_TEM_ and *bla*_SHV_ detected were nucleotide sequenced using the amplification primers.

### Multi locus sequence typing

All randomly selected human *E. coli* isolates (n* = *45), as well as all human ESBL-producing *E. coli* isolates (*n = *5) were analysed by MLST. Among the avian *E. coli* isolates, a simple random selection of 50 ESBL-producing and 50 naïve (lacking resistance phenotypes) isolates were chosen for MLST analysis. If an ESBL-producing isolate was selected from a sample that carried several different ESBL-producing isolates, no further isolates could be selected from that sample.

The MLST scheme by Wirth et al. [23] was applied for analysing seven gene fragments according to the protocols found at http://mlst.ucc.ie/mlst/dbs/Ecoli/documents/primersColi_html as previously described [9]. The MLST allele designations were determined via the online MLST database and all novel sequence alleles and ST designations discovered during this study were provided by the curator of the database (http://mlst.ucc.ie/mlst/dbs/Ecoli). Each new sequence allele was independently verified by the curator of the database by evaluation of a minimum of two nucleotide sequence traces submitted by one of the authors (S.G) along with the information on the new reference strain for the public database. The allele data was analysed by the minimum spanning tree algorithm implemented in Bionumerics v.5.1 (Applied Maths NV) with priority rules set at first link genotypes that have maximum numbers of single-locus variants and then maximal numbers of single-locus variants and double-locus variants. Concatenated nucleotide datasets of the seven genes were used for constructing a neighbour-joining tree based on the number of pair-wise differences among strains using the Pearson coefficient. Forty-seven strains of the ECOR collection previously characterised by MLST were used as phylogroup references [23], [24] The *E. coli* strain Z205/ST125 [23] was included to root the inferred phylogeny. Simpsońs indices of diversity were calculated using the on-line tool at the website http://darwin.phyloviz.net/ComparingPartitions/index.php?link=Home

### Determination of *gyrA* quinolone resistance mutations

All isolates selected for MLST analysis were analysed for the presence of mutations in the positions 248 and 259/260 of the *gyrA* gene sequence because these positions are known to confer fluoroquinolone resistance. We designed the primers gyrAF, *5'* -CGTGTCGTTGGTGACGTAAT-*3'* and gyrAR, 5'-CCACGCGTTTTTCTTTTACC-3' for amplification of 658 nucleotides that were sequenced on both strands using the amplification primers and gyrARseq 5'-AGGAATTTTGGTTGGCATGA-3'.

### Plasmid replicon tying

All samples selected for MLST analysis were also analysed for plasmid replicon types. Plasmids were classified according to incompatibility groups using the PBRT scheme described by Carattoli *et al*.[16]. PCR reactions were performed on the GeneAmp 9700 (Applied Biosystem, Foster City, USA) with JumpStart Taq polymerase (Sigma-Aldrich, Saint Louis Missouri, USA). Amplicons were visualised on 3% ReadyAgarose gels in TBE buffer (Bio-Rad Laboratories Inc., Hercules CA, USA).

### Determination of conjugational transfer of ESBL harboring plasmids

All ESBL-producing isolates selected for MLST analysis were also analysed for conjugational transfer of ESBL resistance. *E. coli* J53 [25] was used as the recipient for detection of conjugation, and mating was carried out overnight at 37°C in LB broth (BD, Le Pont de Claix, France) including sodium azide (100 μg/ml) and cefotaxime (4 μg/ml). Transconjugants were cultured on TSA agar (Oxoid, Basingstoke, England) including above concentrations of sodium azide and cefotaxime. Conjugation was confirmed by testing for presence of ESBL genes by PCR analysis. Plasmid carriage of resistance genotypes was confirmed by conjugational transfer, which was positive for all but one isolate.

## Results

### Antibiotic susceptibility of randomly selected *E. coli* isolates from human and avian samples

Antibiotic susceptibility of randomly selected *E. coli* towards a panel of commonly used antimicrobial agents was assessed in order to receive a general resistance profile of human and avian enterobacteria. From 49 human samples, 45 *E. coli* were isolated, and they showed an overall high susceptibility for most tested agents. The highest resistance frequencies were found for tetracycline and ampicillin, with frequencies of 17.8% for both, followed by nalidixic acid resistance in 6.7% of samples. All isolates were susceptible to cefadroxil, tigecycline and nitrofurantoin, and the remaining tested agents had a resistance frequency of approximately 2 to 4% (mecillinam in 2.2%, and streptomycin, chloramphenicol and trimethoprim -sulphamethoxazole, all in 4.4%, respectively, of isolates).

From the 370 avian samples, 267 *E. coli* were isolated (160 from Concón and 107 from Talcahuano). These showed a general resistance profile similar to that of human samples, with ampicillin resistance in 10.1%, tetracycline resistance in, 8.2%, and streptomycin resistance in 6.0% of the samples. Only nitrofurantoin displayed full susceptibility in all samples, and for cefadroxil there was a 1.1% resistance frequency. Other agents ranged between approximately 0.4 to 5% resistance rate (tigecycline in 0.4%, mecillinam in 1.1%, chloramphenicol in 2.2%, and trimethoprim –sulphamethoxazole in 3.7% of isolates). There was no significant difference in resistance levels between Tapacalhuano and Concon samples or between human and avian samples, as calculated by Fisher’s test.

### Characterization of resistance genes encoding ESBLs in human and avian isolates

Out of the 49 human samples, 6 were found to carry ESBL-producing bacteria; 5 isolates of *E. coli* and one *Enterobacter cloacae* isolate. The isolates contained resistance genes of the group 1 CTX-M type, namely bla_CTX-M-1_ (in two *E. coli* isolates), bla_CTX-M-15_ (two *E. coli* isolates), and bla_CTX-M-30_ (one *E. coli* and one *E. cloacae* isolate). No SHV or TEM type resistance genes were identified.

Out of 372 avian samples, 112 samples carried ESBL-producing bacteria, some samples carried two (*n = *15) or three (*n = *1) phenotypically different isolates. Hence, a total of 129 ESBL-producing isolates were collected, all of which were *E. coli*. 122 isolates contained resistance genes of the group 1 CTX-M type and some of them also contained SHV or TEM type resistance genes (Table 1). Four isolates contained the group 9 CTX-M and two the group 2 CTX-M resistance gene type.

**Table 1:** Distribution of β-lactamase genotypes among 129 ESBL-producing isolates from Franklińs gulls.

No. of isolates	β-lactamase genotypes		
	Group of CTX-M	SHV (presence/absence)	TEM (presence/absence)
101	CTX-M-1	-	-
19	CTX-M-1	-	+
2	CTX-M-1	+	-
2	CTX-M-2	-	-
2	CTX-M-9	-	-
2	CTX-M-9	-	+
1	None detected	+	-

The 50 ESBL-producing *E. coli* isolates from birds included in the MLST analysis showed that isolates with enzymes of the group 1 CTX-M type harbored *bla*_CTX-M-1_ (*n = *39), *bla*_CTX-M-15_ (*n = *8), or *bla*_CTX-M-3_ (*n = *2), and that one isolate with group 9 CTX-M type harbored *bla*_CTX-M-14_ resistance genes. One isolate harbored only *bla*_SHV-12_, while eight CTX-M harboring isolates also harbored the non-ESBL *bla*_TEM-1_. (Table 2)

**Table 2:** CTX-M encoding genes and the presence of *bla*_TEM_ in the MLST-subset of 50 ESBL-producing isolates from Franklińs gulls.

No. of isolates	CTX-M subtype	*bla*_TEM_ (presence/absence)
33	CTX-M-1	-
6	CTX-M-1	+
6	CTX-M-15	+
2	CTX-M-15	-
2	CTX-M-3	-
1	CTX-M-14	+

### Plasmid replicon types of human and avian isolates

Among the 150 (both ESBL and non-ESBL) isolates selected for MLST analysis 102 (68%) contained at least one type of replicon, and the proportion was similar among human (35/50; 70%) and avian (67/100; 67%) isolates (Table 3). In the total material, presence of one or two replicons was common (*n = 57* and *n = 37*, respectively), but isolates with three and four replicons were also found (n = 4 for both). There was a significant difference (P = 0.0006, as calculated by Fisher’s test) in presence of replicons in ESBL harbouring isolates versus non-ESBL isolates among avian samples (42/50 versus 25/50). Due to the small sample sizes (5/5 versus 30/45), this could not be established among human samples (Table 3).

**Table 3:** Total prevalence and the number of distinct replicon types identified in the MLST-subset of isolates from Franklińs gulls and humans.

Sample type	ESBL status	No. of isolates	No. isolates with a given no. of replicon types (percent)				
			≥1	1	2	3	4
Franklińs gulls	ESBL	50	42 (84)	16 (32)	20 (40)	3 (6)	3 (6)
	Non-ESBL	50	25 (50)	15 (30)	10 (20)	0 (0)	0 (0)
Humans	ESBL	5	5 (100)	5 (100)	0 (0)	0 (0)	0 (0)
	Non-ESBL	45	30 (67)	21 (47)	7 (16)	1 (2)	1 (2)
Total		150	102 (68)	57 (38)	37 (25)	4 (3)	4 (3)

Inc FII (n = 47) and Inc I1 (n = 41) were the most frequently detected replicon types among both ESBL/non-ESBL isolates, and human/avian isolates, although IncI1 replicons were more frequent in the ESBL isolates (n = 33) (Table 4). The most common combination of replicons was FII-FIB, present in 18 of 45 isolates with multiple replicons. Most avian *bla*_CTX-M-15_ harboring isolates also displayed the FII-FIB combination, while human *bla*_CTX-M-15_ harboring isolates did not. Further, the FII-FIB replicon was present in several, both avian and human, non-ESBL isolates.

**Table 4:** The diversity of replicon types identified in the MLST-subset of 150 isolates from Franklińs gulls and humans.

Replicon type^a^	No. of isolates containing the indicated replicon types				Total no. of each replicon type
	Franklińs gulls		Humans		
	ESBL	Non-ESBL	ESBL	Non-ESBL	
HI2	1	0	0	0	1
I1	28	1	5	7	41
N	3	0	0	1	4
FIA	4	0	0	0	4
FIB	13	9	0	7	29
Y	7	7	0	4	18
P	1	0	0	0	1
FIC	3	1	0	0	4
A/C	0	0	0	1	1
FII	17	18	0	12	47
K	0	0	0	5	5
B/O	0	0	0	5	5

a The replicon types HI1, X, L/M, W, T, and FIIC were not detected in any of the isolates.

### Conjugational transfer of ESBL harboring plasmids

Conjugational transfer was observed for all but one avian ESBL-harboring isolate. All human ESBL-producing isolates displayed conjugational transfer, although one had a low efficiency and was sensitive to variation in culturing conditions.

### MLST analysis of human and avian isolates

**Figure 1:**
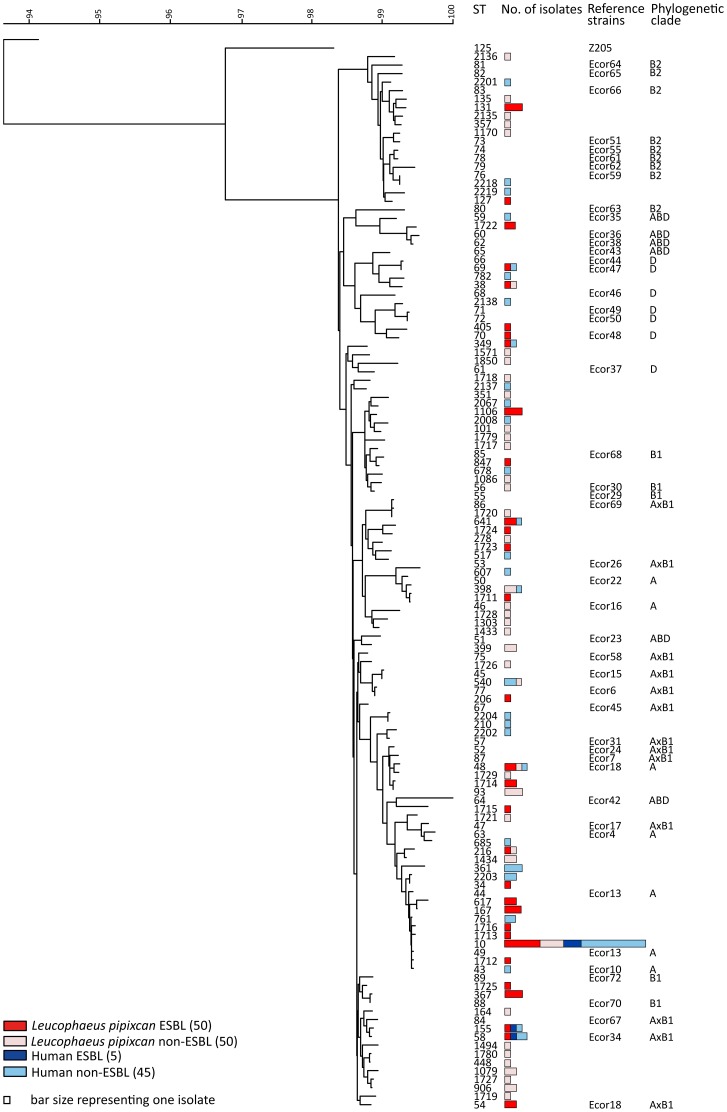
A neighbor joining tree based on seven concatenated gene sequences obtained by MLST. Genetic relationships between different STs and the distribution of the isolates between humans and Franklin's gulls are shown. The new and previously undescribed STs are marked by number 1711 or a higher number. The reference strain Z205 was used to root the tree. Forty-seven strains of the ECOR collection were included in the analysis as references.

In *E. coli* isolated from birds, 67 different STs among the 100 isolates were identified (31 STs among 50 ESBL isolates, 40 STs among 50 non-ESBL isolates). Twenty-three new STs were identified in *E. coli* from birds (10 STs among ESBL isolates, 13 STs among non-ESBL isolates; Figure 1). The most common STs were ST10 (10 isolates), ST1106, ST93, ST131, ST167, ST48 and ST367 (3 isolates in each). ST10 and ST48 were found both among ESBL as well as in non-ESBL *E. coli*.

**Figure 2:**
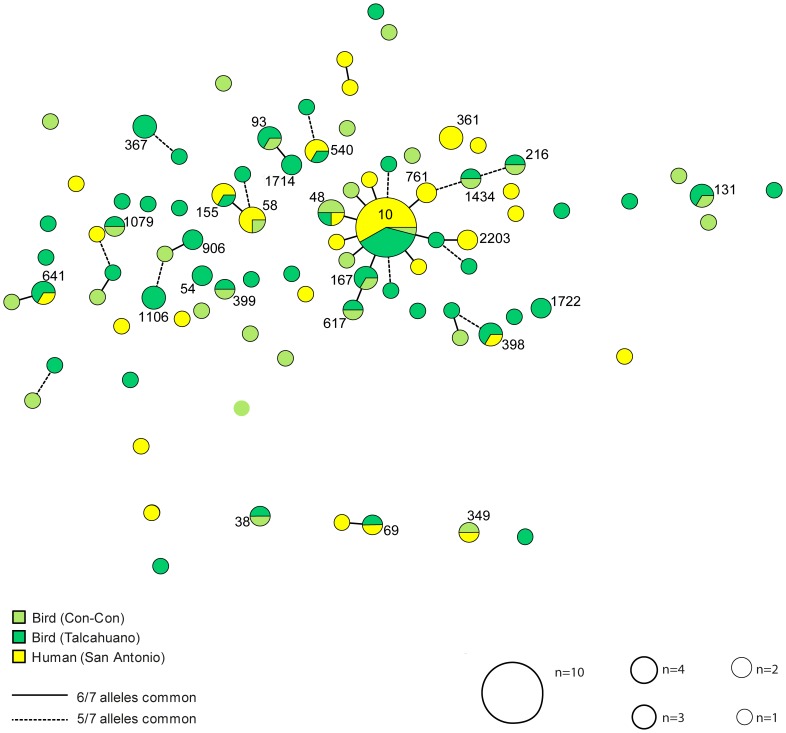
Minimal spanning tree calculated from the allelic differences between the isolates. The tree shows the relationship between the genetic analysis and the geographical source of the isolates.

Among *E. coli* isolated from humans there were 29 different STs among the 50 isolates (3 STs among 5 ESBL isolates; 29 STs among 45 non-ESBL isolates). Eight new STs were identified in *E. coli* from humans (all of them among non-ESBL isolates; Figure 1). The most frequent STs were ST10 (14 isolates), ST58 and ST361, (3 isolates in each) (Figure 1 and 2). Phylogenetic analysis of nucleotide sequences of seven gene fragments showed a diverse population of *E. coli* (Figure 1). Comparison with the reference sequences for *E. coli* of the ECOR-collection showed that all phylogroups were represented in the material. *E. coli* from gulls as well as from humans were spread evenly across the phylogroups and specific STs, with no evident preference of human or avian isolates, respectively. Both ESBL and non-ESBL isolates were found evenly distributed in the phylogenetic tree (Figure 1). Nine of a total of 87 STs were found in both human and avian samples (ST10, ST48, ST58, ST69, ST155, ST349, ST398, ST540 and ST641) (Figure 1). The most common STs were ST10 (24 isolates), ST48 and ST58 (4 isolates each) (Figure 1). There were no obvious phylogeographic patterns, i.e., an analysis by the geographic source of the isolates showed little correlation to the genetic analyses (Figure 2). Some STs contained isolates from all three sampling locations (ST10 and ST48) while several others contained isolates from two of the locations. In Talcahuano, 35% (11/31) of the isolates were ESBL positive. In Con-con, the ratio was 57% (39/69). Some differences in genetic diversity among isolates from gulls and humans were indicated by crude analysis but with overlapping confidence intervals: Simpson’s index of diversity was 0.986 (CI 0.977-0.995) in bird isolates and 0.925 (CI 0.866-0.985) in isolates from humans. Simpson’s index of diversity was 0.991 (0.982-0.999) and 0.976 (0.962-0.991) among resistance naïve avian isolates and ESBL positive avian isolates, respectively.

### Analysis of *gyrA* mediated quinolone resistance

Out of the 50 bird ESBL isolates investigated by MLST, 11 were resistant to NAL and the sequence of the *gyrA* gene was determined. Of the 11 NAL-R isolates, one isolate displayed a resistance mutation in position 83 (Ser83Leu) whereas 10 isolates had two mutations (Ser83Leu, and Asp87Asn). Although several isolates of the same ST exhibited the same mutation (ST131/n = 2, ST167/n = 4, ST617/n = 2), the fact that the same quinolone resistance mutation was found in different STs suggests that the mutations have appeared independently at several time points and is not (only) spread clonally.

Among the five human ESBL-producing *E. coli* none was NAL-R. Among the human non-ESBL isolates three proved to be NAL-R, but none of them had any of the investigated *gyrA* resistance mutations.

## Discussion

We investigated the general level of resistance, with further in-depth analysis of ESBL prevalence in, as well as the phylogeny of *E. coli* in fecal normal flora of humans and Franklin’s gulls in central parts of Chile. There were generally low levels of resistance in randomly selected (one from each sample) human and avian *E. coli* isolates. By selective culturing of for identification of ESBL isolates, however, we found a surprisingly high number of samples harboring ESBL-producing *E. coli* especially in the avian isolates. This illustrates that ESBL-producers in normal flora was not dominant in frequency, as they were rarely picked when cultured unselectively, but that the bacterial population as a whole is well prepared for degradation of cephalosporins. This is to our knowledge the first time the fecal carriage of ESBL has been compared between humans and wildlife from the same area. Unexpectedly, the carriage rate of ESBL-producing bacteria among the gulls 112/372 (30.1%) were more than twice as high as in the human population 6/49 (12.2%). We found mainly ESBLs of *bla*_CTX-M-1_ and *bla*_CTX-M -15_ genotypes that has been extensively described in *E. coli* causing human infections, but also some rare examples of *bla*_CTX_M-3,-14_ and _-30_. A few occurred in combination with SHVs or TEMs, but the larger part was only carrying CTX-M type ESBLs. This confirms the rapid dissemination of different types of CTX-M ESBLs in Chile, both in comparison to the overall general low resistance profile of this material, but also when compared to the low level of SHVs and TEMs, which emerged prior to CTX-M ESBLs in Europe and North America [2], [26]. The high proportion of CTX-M-1 in the avian samples is interesting and could indicate an influx from poultry since CTX-M-1 has been the dominating ESBL in poultry production at least in Europe [27], [28].

Replicon typing identified the FII-FIB replicon combination in a majority of the avian *bla*_CTX-M -15_ harboring isolates, most likely representing a multireplicon contained on a single plasmid. The FII-FIB replicon combination has previously been described to carry *bla*_CTX-M -15_ in *E. coli* from humans [14], and our findings in isolates from birds indicates a zoonotic transfer of these ESBL variants. Interestingly, none of the human *bla*_CTX-M -15_ isolates in this study were of FII-FIB replicon type. Instead *bla*_CTX-M -15_ was found in combination with the I1 replicon type, the overall most common type in this material, mostly found unpaired with other replicon types. This is in line with previous studies describing *E. coli* isolates of both animal and human origin, harboring plasmids of the Inc I1 group and carrying ESBL genes of both *bla*_CTX-M -15_ and *bla*_CTX-M -1_ as well as other genotypes [14]. Further, we found more replicons among avian ESBL isolates than among avian non-ESBL isolates. This is in agreement with a previous study of 127 human uropathogenic *E. coli* isolates lacking phenotypic resistance, showing that such isolates, in contrast to antibiotic resistant isolates, frequently are plasmid naïve and contains few replicon types [29]. Our findings are similar and suggest that although the detected replicon types are present in both ESBLs and non-ESBLs, they occur more frequently in ESBL harboring isolates where they supposedly can facilitate and increase the spread of resistance genes regardless of a human or avian host.

The MLST data of this study support that humans and birds are sharing *E. coli* STs, *i.e.*, there has been exchange of *E coli* between birds and humans. We found nine STs (ST10, ST48, ST58, ST69, ST155, ST349, ST398, ST540 and ST641) present in both avian and human populations (Figure 2). Several of the *E. coli* STs detected in birds have previously been reported as MDR human pathogens including the ability to produce ESBLs. This means that not only commensal flora is shared between humans and birds but also STs with pathogenic potential. We found many STs previously described as major CTX-M carriers [30], including ST10, ST23, ST38, ST46, ST59, ST69, ST101, ST131, ST155, and ST405, of which only ST59 was unique to a human sample. Interestingly, one avian ST38 isolate was found to carry *bla*_CTX-M-1_, although this ST has been reported to display preference for CTX-M group 9 genes [30], [31], [32]. There were many new *E. coli* STs detected in this study from both humans and in birds, and several of the new STs from birds exhibited ESBL-production. In addition, with the caveat that it is difficult to ensure full coverage of the current literature, a number of previously described STs found in our avian material were new to wild animal hosts. Some ESBL-producing STs identified, e.g., ST10, ST48 and ST58 have previously been described in both humans and poultry [33]. For a full list of STs identified in this study, consult Figure 1. It should be noted, that the STs were spread evenly among the isolate populations (four combinations of ESBL or non-ESBL, and human or avian), with no subgrouping present, further supporting the zoonotic behavior of *E. coli*, regardless of ESBL carriage. Considering the potential dissemination of *E. coli* by Franklin’s gulls to nesting places in the central parts of Canada, a recent publication on ESBLs in human clinical isolates from the central Canadian region is of interest since ST10, ST617, ST38, ST131 and ST405 was common in this material ranging from year 2000 to 2010 [34]. Apparently in line with prevalence patterns elsewhere, these STs were all present in our avian isolates and similarly most often carrying *bla*_CTX-M-15._

There was no evidence for local epidemics of any particular *E. coli* ESBL clone among humans or gulls in this material. Rather a scenario emerges where the *E. coli* population as a whole is well prepared for degrading cephalosporins by containing a vast array of different ESBL-genes distributed among many different STs. The fact that the ESBL carriage rate was more than twice as high among the gulls compared to the humans underline that the antibiotic resistance problem must be seen in a large ecological context. We have come to a situation when bacteria both in the environment and in the commensal flora have the capacity to respond to almost any antibiotic used for treatment. Apart from keeping the antibiotic use low, breaking the transmission chains between the environment (e.g. in gulls) and humans must be a priority to hinder the dissemination of resistance.
